# The investigational anti-B7-H3 antibody-drug conjugate vobramitamab duocarmazine exerts anti-tumor activity in vitro and in vivo in pediatric sarcoma preclinical models

**DOI:** 10.1038/s41419-025-08397-z

**Published:** 2026-01-08

**Authors:** Giovanna Bianchi, Fabio Pastorino, Gaia Rolandi, Eleonora Ciampi, Daniela Segalerba, Barbara De Giovanni, Barbara Cafferata, Matilde Balbi, Silvia Ravera, Valerio Gaetano Vellone, Mirco Ponzoni, Chiara Brignole

**Affiliations:** 1 Laboratory of Experimental Therapies in Oncology, IRCCS Istituto Giannina Gaslini, Genoa, Italy; 2 Pathology Unit, IRCCS Istituto Giannina Gaslini, Genoa, Italy; 3 Department of Experimental Medicine, University of Genoa, Genoa, Italy; 4 IRCCS Ospedale Policlinico San Martino, Genoa, Italy; 5 Department of Integrated Surgical and Diagnostic Sciences (DISC), University of Genoa, Genoa, Italy

**Keywords:** Preclinical research, Paediatric cancer

## Abstract

Prognosis for pediatric sarcoma (pSC)-affected patients, especially those with relapsed/refractory disease, is dismal. The available treatment options are unsatisfactory, challenging researchers to address this unmet need. The investigational B7-H3 targeted ADC vobramitamab duocarmazine (vobra duo) showed clinical effectiveness towards several B7-H3-positive adult tumors and pre-clinical efficacy in pediatric neuroblastoma models. Cytotoxicity of vobra duo was evaluated in 2D and 3D models toward pSC cell lines expressing B7-H3, showing a dose-dependent cell viability reduction. Proliferation was assessed by time-lapse single-cell segmentation. Compared to controls, vobra duo resulted in a significant increase in the cell doubling time. AKT/mTOR master effectors of cell proliferation were investigated by phospho-specific western blot assays. A down-modulation of phospho-AKT/ -P70 S6K and -4E-BP1 protein expression was detected in both A204 (rhabdomyosarcoma) and U-2-OS (osteosarcoma) cells, the most treatment-sensitive and resistant cell lines, respectively, suggesting their involvement in vobra duo-mediated anti-proliferative effect. In response to treatment, all cell lines underwent apoptotic cell death. A significant increase in the executioner cleaved caspase-3 was detected, and a partial but significant reversion of apoptotic cell death was noted following pre-treatment with the pan-caspase inhibitor, Q-VD-OP-h. Vobra duo also triggered caspase-independent apoptotic events: i) increased AIF nuclear translocation, ii) increased mitochondrial superoxide production, and iii) the depolarization of mitochondrial membrane potential. In vivo, the effectiveness of vobra duo was assayed by single and repeated intravenous administration in the mouse rhabdomyosarcoma model. The single injection of 3 mg/Kg of vobra duo induced a significant tumor growth delay. Repeated vobra duo doses ameliorated this outcome, reverting rhabdomyosarcorma to rhabdomyoma tumor, by increasing Desmin and Myogenin/Myf-4 differentiation markers expression, and reducing both Ki-67 and CD133. In conclusion, the in vitro and in vivo anti-tumor effects towards pSC highlight the need to extend the investigation to patient-derived preclinical models, to pave the way for clinical translation.

## Introduction

Pediatric sarcomas (pSC) are a large and heterogeneous group of tumors of mesenchymal origin. They account for about 13% of pediatric and young adult cancers, represent approximately 10% of childhood solid tumors, and are responsible for 13% of cancer-related deaths in patients under the age of twenty [1–4]. Based on the primary site of tumor occurrence, pediatric sarcomas are classified into two major categories, soft tissue sarcomas and osseous tumors [1, 5]. In the pediatric population, osteosarcoma and Ewing’s sarcoma are the most common sarcomas of the bone, while rhabdomyosarcoma is the most represented among the wide variety of soft tissue sarcomas [5]. The prognosis for pSC-affected patients is dismal, in particular for those patients with relapsed/refractory disease. If the outcome of localized sarcomas has improved after the introduction of multimodal therapy, it is to be noted that in 20-30% of cases, the disease recurs. Further, pSC often presents metastatic at the onset [5]. So far, the available treatment options are unsatisfactory and new treatment modalities deserve to be investigated.

Antibody-drug conjugates (ADCs), which were developed at the beginning of 2000^th^, to overcome chemotherapy-related side effects, can specifically deliver to cells of interest a potent cytotoxic drug that is coupled to a monoclonal antibody (mAb), directed toward a tumor-associated antigen [6]. They have emerged as a promising tool for treating solid and hematological malignancies [7]. As of 2024, 13 ADCs have been approved by the Food and Drug Administration (FDA) to treat adult tumors [7, 8], many more are in the late stage of approval, and three are expected to receive FDA approval in 2025. Things are different in the *scenario* of pediatric oncology. Indeed, as of the end of 2024, only two ADCs, gentuzumab ozogamicin (Mylotarg) and more recently inozutumab ozogamicin (Besponsa), received the FDA approval to treat CD33-positive acute myeloid leukemia pediatric patients aged 1 month and older, and CD22-positive pediatric patients aged 1 year or older with relapsed or refractory B-cell precursor acute lymphoblastic leukemia. Furthermore, Brentuzumab Vedotin (Adcetris) was approved by the FDA, in combination with chemotherapy, to treat high-risk Hodgkin lymphoma in children and adolescents.

Pediatric sarcomas share with other tumors in childhood and adult the expression of the immunomodulatory antigen B7-H3 (CD276). B7-H3 belongs to the B7 immune checkpoint superfamily; it is endowed with immune-modulatory properties, and, although still controversial, even because of the yet unknown receptor, it seems to be involved in immunosuppression [9]. Noteworthy, B7-H3 is overexpressed in adult and childhood malignancies by tumor cells, tumor-associated vessels [10] and tumor-associated stromal cells [11], while having a restricted expression in healthy organs, due to a fine post-transcriptional regulation [11]. Being a good target for immunotherapeutic approaches, in the last decade, it has gained great attention, and several lines of research have focused on deeply and univocally understanding its biological function and developing treatment modalities to target it (*eg* cell-based therapies, mAbs, bispecific mAb, ADCs, and radio-conjugated mAb) [12]. Clinical trials targeting B7-H3, through several approaches including ADCs, CAR-T cells, bispecific Abs in a variety of adult solid tumors and less represented pediatric malignancies, have been opened, and some of those are still recruiting patients [13–16].

Vobramitamab duocarmazine, (vobra duo, also known as MGC018; MacroGenics, Inc., Rockville, MD, USA) is an investigational duocarmycin-based ADC directed against the B7-H3 antigen that showed clinical anti-tumor activity in adult cancers (*eg* non-small cell lung cancer, metastatic castration-resistant prostate cancer, melanoma, triple-negative breast cancer) [17, 18] as well as preclinical activity in neuroblastoma [19] and, as proof of functionality, in other pediatric tumors, including pSC [20].

This study investigated the anti-tumor activity and, for the first time to our knowledge, the underlying mechanisms of action of vobra duo in pSC, providing evidence of its effectiveness in preclinical in vitro and in vivo models.

## Materials And Methods

### Cell lines and B7-H3 expression

A panel of pediatric sarcoma (pSC) cell lines, including rhabdomyosarcoma (A204) and osteosarcoma (HOS, MG-63 and U-2-OS), was purchased from the Cell Bank Interlab Cell Line Collection (ICLC) of the IRCCS Ospedale Policlinico San Martino.

Cells were grown in DMEM high glucose (Euroclone), supplemented with 10% of heat inactivated fetal bovine serum (FBS, Gibco), 50 IU/mL penicillin G, 50 µg/mL streptomycin sulphate, and 2 mM L-glutamine (Euroclone). Cell lines were periodically tested for mycoplasma contamination by polymerase chain reaction (PCR) assay, characterized by cell proliferation and morphology evaluation, and authenticated by multiplex short-tandem repeat profiling by BMR Genomics (Padova, Italy).

Surface expression of B7-H3 was determined by flow cytometry analysis (FCM) on a Gallios Flow Cytometer (Beckman Coulter). Briefly, cells (2 × 10^5^ cells/tube) were washed with phosphate-buffered saline (PBS) supplemented with 2 mM EDTA and 1% FBS and then incubated with anti-B7-H3 mAb (see Antibodies and ADC section) at a final concentration of 1 µg/mL in a staining volume of 100 µL for 30 minutes (min) at 4°C. Cells were also stained with an isotype matched mAb, as control. Cells were then washed with PBS, (2 mM EDTA, 1% FBS), and the expression of B7-H3 was reported as mean ratio fluorescence intensity (MRFI), defined as the ratio of the MFI of cells labeled with anti-B7-H3 mAb over the MFI of cells stained with the isotype-matched control mAb.

### Cytotoxicity and apoptosis assays

Cell lines were seeded in 96-well plates (1–2 × 10^3^ cells/well) and treated on the day after seeding, with escalating concentrations of vobra duo (MacroGenics, Inc.) in continuous for 96 hours (h). Multicellular tumor spheroids (MCTS), an in vitro model consisting of scaffold-free, self-assembled spherical cell aggregates, were generated by seeding A204, HOS, MG-63, and U-2-OS (1–2 × 10^3^ cells/well) in black ultra-low-attachment (ULA) 96-well plates (Corning, Steuben, NY, USA), with spheroids typically forming after three days of culture. MCTS of A204, HOS, MG-63, and U-2-OS were treated with vobra duo, in continuous for 96 h, as above. Each experimental condition was carried out in quadruplicate wells. Monolayer-cultured cell viability was determined by the MTS colorimetric assay, according to the manufacturer’s instructions (CellTiter 96® Aqueous One Solution Cell Proliferation Assay, Promega Italia, Milano, Italy). The optical density (OD) of colored formazan, produced by viable cells, was determined by the GloMax Discover (Promega) microplate reader. The viability of MCTS was assessed by the CellTiter-Glo® 3D Cell Viability Assay (Promega) and recorded as relative luminescence units (RLU) on the GloMax Discover instrument.

To assess apoptotic cell death, cell lines were seeded in 6-well plates and treated, the day after seeding, with escalating concentrations of vobra duo, for 24 to 72 h. Apoptotic cell death was assessed by FCM using the annexin V-FITC kit from Beckman Coulter (Brea, CA, USA), following the manufacturer’s instructions. Gallios Instrument was used for the data acquisition; the results were analyzed thanks to Kaluza software. In some experiments, cells were pre-treated with the pan-caspases inhibitor Q-VD-OPh (Sigma-Aldrich, St. Louis, MO, USA) at 30 µM concentration for 30 min and then treated with vobra duo for 48 h.

The expression of cleaved caspases -3 and -7 in response to vobra duo treatment was also determined by using specific primary antibodies (see Antibodies and ADC section), according to Cell Signaling Technology protocols.

### Evaluation of caspase-independent apoptotic events

To investigate caspase-independent apoptotic events, pSC cell lines were seeded in: i) 6-well plates (8 × 10^4^- 3 × 10^5^) to measure mitochondrial ROS production and mitochondrial membrane potential changes, and ii) in 8-well chamber slides (2 × 10^4^ A204 and 1 × 10^4^ HOS, MG-63 and U-2-OS) to investigate Apoptosis Inducing Factor (AIF) expression. The day after seeding, cells were treated with vobra duo (0.8 μg/mL for A204, 8 μg/mL for HOS and MG-63 and 16 μg/mL U-2-OS) for 48 h.

#### AIF immunofluorescence staining

For immunofluorescence analysis, at the end of the treatment, cells were fixed with 4% PFA and permeabilized for 30 min with 0.1% Triton X-100. Then, they were placed in blocking solution (2% BSA) for 30 min and exposed to primary anti-AIF antibody (see Antibodies and ADC section) for 1 h at room temperature (RT), followed by incubation with secondary antibody (see Antibodies and ADC section) for 30 min at RT in the dark. The specificity of immunoreactivity was checked by omission of the primary antibody. Nuclei were counterstained with DAPI (Vectashield, Vector Laboratories, Burlingame, CA). Images were then analyzed by an Axioplan Imager M2 microscope, software AxioVs40 version 4.8.2.0 (Zeiss, Oberkochen, Germany) and colocalization of AIF and DAPI fluorescence was analyzed with Fiji-Image J software.

#### Evaluation of mitochondrial ROS production

Mitochondrial superoxide production was assessed by using the MitoSOX™ Mitochondrial Superoxide Indicators (Invitrogen). Briefly, at the end of the treatment, cells were harvested, washed, and stained with MitoSOX™ Red (MRS) at the final concentration of 5 µM in PBS 1% FBS, 15 min at 37 °C. After incubation, cells were washed twice and then analyzed by FCM.

#### Mitochondrial membrane potential changes measurement

Mitochondrial activity was measured with MitoProbe JC-1 Assay Kit (#M34152, Invitrogen, Italy) according to the manufacturer’s instructions. Briefly, at the end of the treatment, cells were collected (2 × 10^5^ cells/tube), washed, and suspended in 200 µL PBS at RT. Cells were pre-incubated or not with the oxidative phosphorylation inhibitor, carbonyl cyanide 3-chlorophenylhydrazone (CCCP) (50 µM) for 10 min at 37°C and then stained with JC-1 probe at the final concentration of 2 µM, for 30 min at 37°C. After a final washing step, fluorescence was measured by FCM. JC-1 is a probe that accumulates within the cell in a mitochondria membrane potential-dependent fashion. It assembles red fluorescent aggregates (590 nm; red) in polarized mitochondria while it monomerizes in depolarized mitochondria, emitting a green fluorescence (527 nm; green) [21].

### Aerobic ATP synthesis assay

FoF₁-ATP synthase activity was determined in 1×10⁵ cells resuspended in PBS containing 0.6 mM ouabain (to inhibit Na⁺/K⁺-ATPase) and 0.25 mM diadenosine-5′-pentaphosphate (adenylate kinase inhibitor; Merck, Darmstadt, Germany). After a 10-min incubation, respiration was stimulated with either 10 mM pyruvate plus 5 mM malate or 20 mM succinate. ATP synthesis was quantified using a luminometer (GloMax® 20/20, Promega Italia, Milan, Italy) and a luciferin/luciferase-based bioluminescence assay kit (CLS II, #11699695001; Roche, Basel, Switzerland) after the addition of 0.1 mM ADP. Luminescence was measured every 30 seconds over a 2 min period [22].

### Cellular energy status evaluation

The cellular energy status was measured as the ratio between the intracellular contents of ATP and AMP. Intracellular levels of ATP and AMP were determined using an enzyme-coupled spectrophotometric assay monitoring the reduction of NADP⁺ and the NADH reduction at 340 nm, respectively, according to previous report [23].

### Anaerobic glycolysis yield evaluation

The efficiency of anaerobic glycolysis was calculated as the ratio between the measured lactate in the growth medium and the theoretical yield corresponding to the double of consumed glucose, assuming that complete anaerobic conversion of one glucose molecule results in the formation of two lactate molecules. Glucose and lactate concentration in the growth medium have been assayed spectrophotometrically, following the NADP⁺ and the NAD⁺ reduction at 340 nm, respectively as reported in previous report [24] and data are normalized on the cell number.

### Proliferation and morphology assessment

In order to determine whether vobra duo treatment affects cell proliferation and cell morphology, pSC cells were seeded in 96-well plates (1–2 × 10^3^ cells/well) and treated in continuous for 72 h with vobra duo (0.8 μg/ml for A204, and 10 μg/mL for MG-63, HOS and U-2-OS). A time-lapse single-cell segmentation acquisition was carried out using the Livecyte instrument (PhaseFocus), which, thanks to the ptychography technology, generates high-contrast, label-free images, allowing for robust individual cell segmentation during long-term time-lapse. Cell doubling time (Cdt) and cell morphology (*ie* cell area) were analyzed using the PhaseFocus Cell Analysis Toolbox (CAT) version 3.8.1. To dissect the pathway involved in the inhibition of cell proliferation, the pan- AKT inhibitor AZD 5363 was used. Moreover, IGF-1, known to be able to revert the down-modulation of the PI3K/AKT pathway [25, 26], was also used. Cells were seeded in 96-well plates, as described above, and treated with vobra duo, AZD 5363 (5 µM), and IGF-1 (30 nM) alone and in combination, for 72 hours. Cell doubling time was assessed as reported above, through a time-lapse single-cell segmentation acquisition.

In some experiments, pSC cell lines were seeded in 4-well chamber slides (10−15 × 10^3^/well) and treated the day after with vobra duo. After 72 h of treatment, cells were stained with rhodamine-labeled phalloidin (Phalloidin-Atto 532, Sigma-Aldrich) and counterstained with DAPI. Cells were then analyzed by a fluorescence microscope, as already described above.

### Western blot assay

A204 and U-2-OS cell lines were seeded on 75 cm^2^ cell culture flasks (1 × 10^6^ and 6 × 10^5^ respectively) and treated with vobra duo (0.8 μg/ml for A204 and 1.5 μg/ml for U-2-OS) for 72 hours. At the end of the treatment, cells were collected and lysed in RIPA Lysis and Extraction Buffer (ThermoScientific #89900) with a complete protease inhibitor cocktail. Cell lysates were subjected to centrifugation at 15,000 g at 4 °C for 10 min. Supernatant protein concentration was calculated using the BCA assay (Euroclone, #EMP014250) following the manufacturer’s instructions. Equal amounts of protein (30 μg) were separated onto gradient 4–15% Mini-PROTEAN® TGX™ Precast Protein Gels, (Biorad Laboratories Inc., #4561083) and then transferred to nitrocellulose membrane with Trans-Blot Turbo system (Biorad, #1704159) and analyzed by Western blotting. Proteins were detected using antibodies listed in the Antibodies and ADC section. The proteins were visualized by chemiluminescence using the Clarity Western ECL Substrate (Biorad, #1705061). Chemiluminescence was monitored using the Molecular Imager ChemiDoc XRS System. Images were analyzed with ImageLab software (Biorad). Bands were analyzed as Region-of-Interest (ROI), normalized against the GAPDH loading control. The molecular weight of the proteins (based on the Precision Plus Protein WesternC Standards, Biorad #161-0374) was calculated using the band analysis program of the software Quantity one 4.6 of the Molecular Imager ChemiDoc XRS System.

### Antibodies and ADCs

The following antibodies were used for:

i) cytofluorimetric analysis

- PE-conjugated mouse IgG1 anti-B7-H3 mAb (BioLegend, San Diego, California, USA, #331606) - PE-conjugated isotype matched mAb (Becton, Dickinson, Milano, Italy, #554680)

- Alexa Fluor 488-conjugated Cleaved Caspase-3 (Asp175) (D3E9) Rabbit mAb (Cell Signaling, Danvers, Massachusetts, USA, #9603)

- PE-conjugated Cleaved Caspase-7 (Asp198) (D6H1) Rabbit mAb (Cell Signaling, #42524)

ii) western blot assay

- Rabbit monoclonal antibody (mAb) anti-Phospho-Akt (Ser473) (Cell Signaling, #4058)

- Mouse mAb anti-Akt (Cell Signaling, #2966S)

- Mouse mAb anti-Phospho-P70 S6 Kinase (Thr389) (Cell Signaling, #9206)

- Rabbit mAb anti-P70 S6K (Merk Millipore, Darmstadt, Germany, #07-402)

- Rabbit mAb anti-Phospho-4E-BP1 (Thr37/46) (Cell Signaling, #2855)

- Rabbit mAb anti-4E-BP1, (Cell Signaling, #9644S)

- Mouse mAb anti-GAPDH (Santa Cruz Biotechnology, Dallas, Texas, USA, #sc-365062)

- Anti-rabbit IgG, HRP-linked Antibody (Cell Signaling, #7074)

- Anti-mouse IgG, HRP-linked Antibody (Cell Signaling, #7076)

iii) immunofluorescence staining

- Rabbit mAb anti-AIF (Cell Signaling, #5318)

- Alexa Fluor 488 goat anti-rabbit IgG (ThermoScientific, #A-11008)

#### ADC

MGA017, the unconjugated humanized anti-B7-H3 mAb precursor of the ADC, was produced at MacroGenics (Rockville, Maryland, USA) and then conjugated to vc-seco-DUBA at Byondis, the Netherlands, to form vobra duo (MGC018) [27].

### Sarcoma mouse models

Five-week-old female athymic nude-Foxn1nu (nu/nu) mice (Envigo, Bresso, Italy) were housed under specific pathogen-free conditions. In accordance with the “3Rs policy”, experiments were reviewed and approved by the Animal Welfare Body (OPBA) of IRCCS Ospedale Policlinico San Martino and by the Italian Ministry of Health (n. 539/2024-PR). For the subcutaneous rhabdomyosarcoma murine model, A204 cell line (5 × 10^6^ cells in 200 µL of culture medium) was injected under the skin in the right flank of 5 week-old mice, as previously described [28]. Seven days after tumor cell inoculation, when tumors were palpable, mice were randomly divided into four groups of 10 animals each: the control (CTR) group received PBS; the 1 Dose, 2 Doses and 3 Doses groups were treated with 3 mg/kg of vobra duo, intravenously administered once a week for one, two or three weeks, respectively. Animals were monitored twice a week, and tumor masses were measured with a caliper. The tumor volume permitted by the ethics committee is 1.5-2 cm^3^ in size. In this work, the maximal tumor size was not exceeded. Tumor volume was determined by using an external caliper, and it was calculated using the following equation: tumor volume (mm^3^) = length × (width^2^) × π/6, expressing length and width in mm. Survival was ascertained by a humane endpoint. If signs of discomfort or poor health arose (*eg* abdominal dilatation, dehydration, paraplegia, >15% weight loss), mice were sacrificed by CO_2_ inhalation. The day of euthanasia was recorded as the day of death. In all the experiments, mouse body weight and general physical status were recorded weekly and 24 h before and after each treatment.

### Histologic analysis of mouse rhabdomyosarcoma subcutaneous tumors

Histologic, immunohistochemical and immunofluorescence evaluations of tumor tissues were carried out: i) on day 24 (*ie* 10 days after the third vobra duo treatment, for Ki-67 and CD133 expression markers, and ii) after 24 h from the first dose of vobra duo for TUNEL assay, respectively. Briefly, mice were anesthetized with xylazine and sacrificed by CO_2_ inhalation. Tumor masses were collected and then processed for paraffin embedding.

#### Immunohistochemistry

Immunohistochemical (IHC) staining was carried out on formalin-fixed, paraffin-embedded tumor sections using the fully automated Ventana BenchMark XT platform (Ventana Medical Systems Inc., Tucson, AZ, USA), according to the manufacturer’s protocols. Antigen retrieval was performed with a citrate-based buffer (pH 6.0) at 90 °C for 30 minutes. Sections were then incubated with the primary antibodies for 1 hour at 37 °C. The following antibodies were used: anti-Ki-67 (monoclonal rabbit, clone SP6, Ventana, pre-diluted) and anti-CD133 (monoclonal, clone AC133, Miltenyi Biotec, pre-diluted). Detection was achieved using the UltraView Universal DAB Detection Kit (Ventana Medical Systems Inc.), and counterstaining was performed with Gill’s Modified Hematoxylin for 8 minutes at room temperature, followed by 4 minutes of Bluing Reagent (Ventana Medical Systems Inc.). IHC staining was also carried out for Desmin and Myogenin/Myf-4, using prediluted primary antibodies provided by the manufacturer (anti-Desmin, clone DE-R-11, and anti- Myogenin/Myf-4, clone F5D). Antigen retrieval, staining, and counterstaining were carried out automatically according to the manufacturer’s protocols, using the ultraView Universal DAB Detection Kit (Ventana, Roche). Appropriate positive and negative controls were included in each run. Stained slides were evaluated by light microscopy to assess the extent and intensity of marker expression.

#### Immunofluorescence, terminal deoxynucleotidyl transferase-mediated dUTP nick end labeling assay

Terminal deoxyribonucleotide transferase–mediated nick-end labeling (TUNEL) analysis was performed with the in situ Cell Death Detection kit (Roche) according to the manufacturer’s instructions. Briefly, sections were incubated with 100 μL of terminal deoxynucleotidyl transferase-mediated dUTP nick end labeling (TUNEL) reaction mixture, which contained terminal deoxynucleotidyl transferase and FITC-labeled dUTP, in a humidified atmosphere for 1 h at 37 °C in the dark. The slides were rinsed and mounted with coverslips using Vectashield mounting medium with DAPI. Cells were analyzed with a fluorescence microscope as described above. TUNEL-positive signals were determined by counting at least five randomly selected fields.

### Statistics

All the in vitro experiments were performed at least three times, with similar results. Differential findings among the experimental conditions were determined by one-way analysis of variance, with Tukey’s multiple comparison test, using GraphPad Prism V.5 (GraphPad Software, San Diego, California, USA). In the case of experiments comparing control *vs* vobra duo, the statistical difference was calculated by using an unpaired t-test with Welch’s correction. Survival curves were drawn as Kaplan-Meier Cumulative Proportion Surviving graphs and corresponding *p*-values were calculated by using the log-rank (Mantel-Cox) test. Asterisks indicate the following p-value ranges: *p < 0.05, **p < 0.01, ***p < 0.001. The number of animals used was the minimum necessary to achieve the study objectives, based also on prior experiences. As approved by the Animal Welfare Body (OPBA) of IRCCS Ospedale Policlinico San Martino and by the Italian Ministry of Health (n. 539/2024-PR), all in vivo functional experiments were performed with 10 mice per experimental group. This number was calculated considering reductions in tumor growth of at least 35% in the treated groups compared to controls, a type I error of *α* = 0.05, a statistical power of 80%, and a standard deviation of 25%.

## Results

### Vobra duo exerts anti-tumor activity toward pSC cell lines in monolayer and MCTS models

**Fig. 1:**
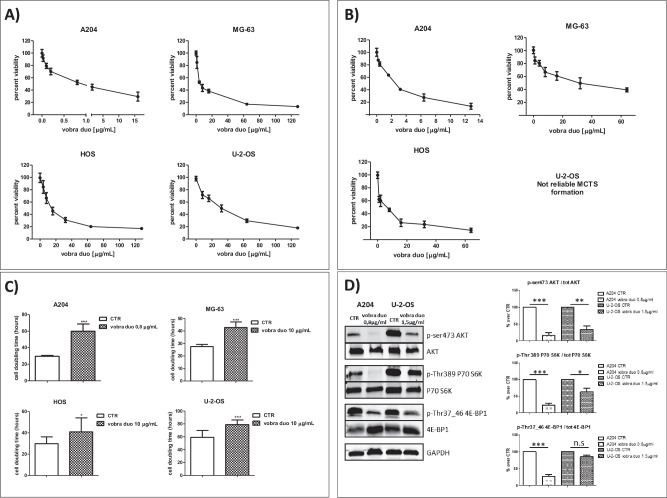
Vobra duo exerted cytotoxic activity and inhibited proliferation and survival toward pSC tumor cell lines. pSC cell lines cultured as monolayer (**A**−**D**) or as multicellular tumor spheroids (**B**) were exposed to vobra duo treatment for 96 h. At the end of treatment, cell viability of monolayer cultured cells (**A**) was measured by the MTS assay, while the CellTiter-Glo® 3D Cell Viability Assay was used for MCTS (**B**). A time-lapse single-cell segmentation analysis was carried out by using a pticography technology-based instrument. The results of single-cell segmentation are reported in terms of cell-doubling time (**C**). Western blot analysis for the AKT/mTOR pathway showed both the total and the phosphorylated protein levels of AKT, P70 S6K and 4E-BP1 in A204 and U-2-OS cell lines, treated or not with sublethal doses of vobra duo for 72 h. GAPDH expression was analyzed as housekeeping gene. A representative experiment, of at least three, has been reported (**D**). The graphical quantification of phospho-AKT, phospho-P70S6K and phospho-4E-BP1 normalized by total AKT, P70 S6K and 4E-BP1 reported in (**D**). Results are expressed as mean ± SD of at least three independent experiments. Significance is referred to the controls.*** *p* < 0.001, ** *p* < 0.01, * *p* < 0.05 (**D**). Results are expressed as mean ± SD of at least three independent experiments. In every experiment performed in 96-well plates, each experimental condition was carried out in quadruplicate. Significance is referred to the controls. OD: optical density.

All the pSC cell lines used in this study, subjected to validation of their surface B7-H3 expression, showed a unimodal expression of the B7-H3 antigen (Supplementary Fig. 1). The administration of vobra duo to pSC cell lines resulted in a dose-dependent reduction of cell viability in both monolayer (Fig. 1A) and MCTS models (Fig. 1B). The sensitivity to a 96 h-treatment was dissimilar, with the A204 rhabdomyosarcoma cell line being the more treatment-sensitive (IC50 = 0.27 µg/mL, Table 1). Osteosarcoma cell lines were less sensitive to the treatment, with IC50 values ranging between 4.4 to 18.5 µg/mL of vobra duo (Table 1). In the MCTS model, all the cell lines showed a similar behavior, with IC50 values ranging between 1.3 to 4,9 µg/mL (Table 1**)**. It is to be noted that the U-2-OS osteosarcoma cell line was excluded from the analysis because their MCTS formation inter-experiments was not reliable, and likely, it could have invalidated the result.

**Table 1:** The IC50 values were calculated for both the culture models tested, by using GraphPad Prism V.5 software.

	Monolayer model			MCTS model		
Cell lines	IC50	R2	95% CI	IC50	R2	95% CI
**A204**	0,27 µg/mL	0,96	0,25 to 0,31 µg/mL	1,7 µg/mL	0,83	1,4 to 2,1 ug/mL
**MG-63**	4,4 µg/mL	0,95	3,8 to 5,1 µg/mL	4,9 µg/mL	0,76	3,3 to 7,3 μg/mL
**HOS**	10,1 ug/mL	0,95	9,312 to 10,93 μg/mL	1,3 µg/mL	0,84	0,9 to 2 µg/mL
**U-2-OS**	18,5 μg/mL	0,89	15,9 to 20,5 µg/mL	NA	NA	NA

*CI* confidence interval.

Vobra duo treatment, besides affecting cell viability, also determined a reduction in cell proliferation. All the cell lines tested showed a statistically significant increase in cell doubling time (Fig. 1C). A204 and U-2-OS cell lines, respectively, displayed the greatest and the lowest sensitivity to vobra duo in terms of viability and cellular proliferation (Fig. 1A,C and Table 1), and for this reason they were selected to measure the expression levels of some of the main master regulator proteins of cellular proliferation and survival (AKT, P70 S6K, and 4E-BP1) involved in the AKT/mTOR pathway. Our findings revealed a strong decrease in the phosphorylation levels of AKT, an upstream modulator of mTOR, both in A204 and U-2-OS cells, following vobra duo treatment (% of phospho- AKT / total AKT ratio over CTR: A204 16.6%, *p* < 0.001; U-2-OS 33.6%, *p* < 0.01) (Fig. 1D). Moreover, vobra duo treatment caused a great decrease in the phosphorylation levels of two downstream players of the AKT/mTOR axis, phospho-P70 S6K (% of phospho-P70 S6K / total P70 S6K ratio over CTR: A204 23%, *p* < 0.001; U-2-OS 63% p < 0.05) and phospho-4E-BP1 (% of phospho-4E-BP1 / total 4E-BP1 ratio over CTR:A204 27.3%, *p* < 0.001; U-2-OS 72% n.s.) (Fig. 1D). Altogether, our results suggest that vobra duo exerts a pronounced anti-tumor effect toward rhabdomyosarcoma and osteosarcoma cell lines, by impairing tumor growth, proliferation and survival and by increasing tumor cell death.

Experiments carried out to determine the involvement of the mTOR/AKT pathway in the aforementioned anti-proliferative effects, demonstrated that the combination of vobra duo and the pan-AKT inhibitor AZD 5363 determined a statistically significant increase in cell doubling time when compared to both agents administered alone (Supplementary Fig. 2A). The pre-treatment of the cells with IGF-1 was unable to revert the effect of vobra duo toward A204 cell line, which indeed does not express IGF-1 R [29]. On the other hand, IGF-1 was able to significantly, although partially, revert the effect of vobra duo in terms of cell doubling time on U-2-OS and MG-63 (Supplementary Fig. 2B), which express IGF-1 R, as already reported [29].

**Fig. 2:**
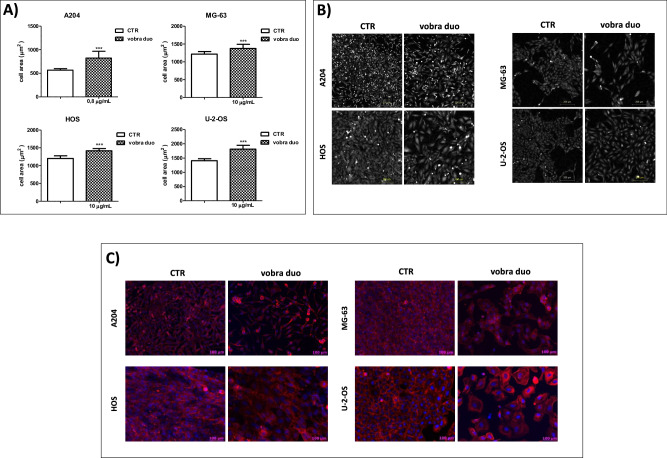
Vobra duo drove pSC cells to undergo morphological changes. A time-lapse single-cell segmentation analysis was carried out by using a pticography technology-based instrument. The results of single-cell segmentation are reported in terms of cell area increase (**A**). Representative images captured after 96 h of treatment with vobra duo, during a time-lapse acquisition scan, performed by Livecyte instrument. Pticography technology allowed generating high-contrast label-free images. Acquisition scans were carried out by a 10x xapo objective (**B**). pSC were grown in 8-well chamber slides and then treated with vobra duo for 96 h. Representative images of vobra duo-treated and untreated cells, after being stained with anti-phalloidin PE mAb and then counterstained with DAPI. A 40x magnification was used (**C**).

The above effects on cell viability and proliferation were also associated with morphological changes, with cells appearing bigger than the untreated ones, as confirmed by a statistically significant increase in cell area (Fig. 2A, B). Morphological observations revealed a flattened shape with cytoskeleton reorganization (Fig. 2 B, C). The increased cell dimension, associated with an enhanced cell complexity, were also evidenced by the augmented physical parameters (forward scatter, FSC and side scatter, SSC) detected in all the flow cytometry analyses performed throughout this study (see the FSC *vs* SSC dot plots of Supplementary Fig. 3 as a representative example).

### Vobra duo induces caspase-dependent and –independent apoptotic cell death

**Fig. 3:**
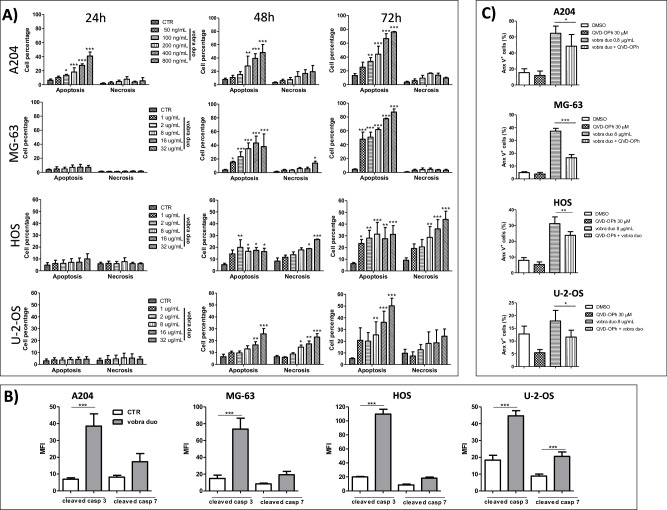
Vobra duo partially induced caspase-dependent apoptosis. **A** pSC cells were treated with increasing concentrations of vobra duo, for 24, 48 and 72 h, and apoptosis was detected by the use of the Annexin V assay. Histograms show the percentage of apoptotic and necrotic cells. **B** Cells were treated with vobra duo at the concentrations indicated for 48 h. At the end of treatment, the activation of caspases 3 and 7 was determined by the detection of their cleaved form. Histograms represent the mean ratio fluorescence intensity (MRFI) of vobra duo-treated cells compared to control untreated ones. **C** Cells were pre-treated with the pan-caspase inhibitor Q-VD-OPh, before being exposed to vobra duo. Apoptosis was evaluated after 48 h of treatment. Data are expressed as mean ± SD of at least three independent experiments. Significance is referred to controls.***p < 0.001, **p < 0.01, *p < 0.05.

**Fig. 4:**
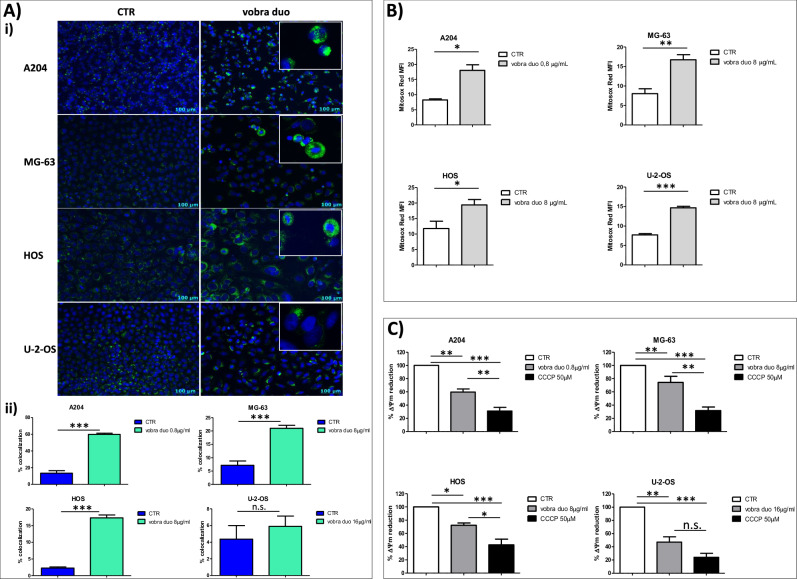
Vobra duo induced mitochondria-dependent apoptosis. **A** i) AIF release and translocation from mitochondria to the nucleus, imaged by microscopy in pSC cell lines exposed to vobra duo (0,8 μg /mL for A204, 8 μg/mL for HOS and MG-63 and 16 μg/mL U-2-OS) continuously administered for 48 h hours. AIF (green) and nuclear staining (DAPI, blue) fluorescence have been shown. Original magnification 20× of a representative field for each condition was reported. **ii**) Graphical quantification of AIF and DAPI fluorescence co-localization. **B** pSC cells were treated with vobra duo at the concentrations indicated for 48 h. At the end of treatment, cells were harvested and incubated with the Mitosox Red indicator. Samples were run on a Gallios flow cytometer, and red fluorescence was collected. Results are expressed as mean fluorescence intensity (MFI) ± SD of three independent experiments. Significance refers to controls. *** *p* < 0.001, ** *p* < 0.01, * *p* < 0.05. **C** Quantitative representation of the percentage of depolarization, obtained by the normalization of the red/green JC-1 MRFI of treated *vs* control pSC cell lines.

The mechanisms responsible for cell viability reduction, observed in response to vobra duo treatment, were investigated. All the pSC cell lines tested underwent significant dose- and time-dependent apoptotic cell death, as assessed by the Annexin V assay, although following a different kinetic (Fig. 3A). More in-depth investigations were performed. The detection of significantly increased levels of the executioner cleaved caspase-3, after vobra duo treatment, further supported apoptotic cell death (Fig. 3B). Moreover, increased levels of the executioner cleaved caspase-7 were also detected in the U-2-OS cell line (Fig. 3B). Surprisingly, the pre-treatment with the pan-caspase inhibitor Q-VD-OP-h, only partially rescued the percentage of apoptosis to the control levels (Fig. 3C). Previous reports provide evidence that in stress conditions, such as oxidative stress or DNA damage, mitochondria can release caspase-independent apoptosis mediators, such as, Apoptosis-Inducing Factor (AIF), which translocates to the nucleus promoting DNA fragmentation and cell death [30, 31]. In order to investigate the effect of vobra duo on AIF functionality in pSC, tumor cell lines were treated with vobra duo for 48 h and AIF expression was investigated by immunofluorescence analysis. Vobra duo treatment, in comparison to untreated cells, caused in A204, HOS and MG-63 cell lines, a significant change in AIF distribution within the cells, as demonstrated both by the peri-nuclear displacement and by the significant increase of AIF/DAPI fluorescence co-localization (green/blue, respectively; Fig. 4A i-ii**)**. In the U-2-OS cell line, AIF protein appeared to have the tendency to migrate into the nucleus but the phenomenon was not significant (Figure 4Ai-ii) and this was in line with the increased expression of both cleavedcasapase-3 and -7 and the capability of pan-caspase inhibitor Q-VD-OP-h pre-treatment, in U-2-OS, to almost completely rescue the percentage of apoptosis to the control levels (Fig. 3C). To corroborate mitochondrial stress induction in pSC by vobra duo treatment, we investigated mitochondrial superoxide production and membrane potential (ΔΨm) depolarization. As reported in Fig. 4B, A204, HOS, MG-63 and U-2-OS cell lines treated with vobra duo for 48 h, showed a significant increase in mitochondrial superoxide production in comparison with control cells. Moreover, flow cytometry analysis of JC-1 dye fluorescence revealed that vobra duo induced a significant reduction of the ΔΨm (Fig. 4C and Supplementary Fig. 3) in all tested pSC cells, in comparison with untreated ones. After vobra duo treatment, both green and red fluorescence increased in comparison to control cells. This effect could be due to the observed increase in cell size and complexity in treated samples, as indicated by FSC and SSC parameters in cytofluorimetric assessment (Supplementary Fig. 3), which may have influenced JC-1 mean fluorescence intensity (MFI) measurements. Nevertheless, the red/green MFI ratio (MRFI) was significantly down-modulated in pSC treated cells, suggesting a partial mitochondria membrane depolarization in comparison with untreated cells. In order to account for inter-experimental variability, the red/green MRFI of treated samples was normalized *vs* control cells, offering a percentage of depolarization. In addition, vobra duo treatment induced a time-dependent impairment of aerobic ATP synthesis through the FoF₁-ATP synthase in both A204 and U-2-OS cells, leading to a decrease in cellular energy status and a metabolic shift toward anaerobic glycolysis as an attempt to compensate for the altered aerobic metabolism (Supplementary Fig. 4).

Together, these results demonstrated that vobra duo affects cell viability by increasing caspase-dependent and –independent apoptotic cell death and by leading to reduced mitochondria functionality and stability.

### Anti-tumor activity of vobra duo in pSC mouse model

**Fig. 5:**
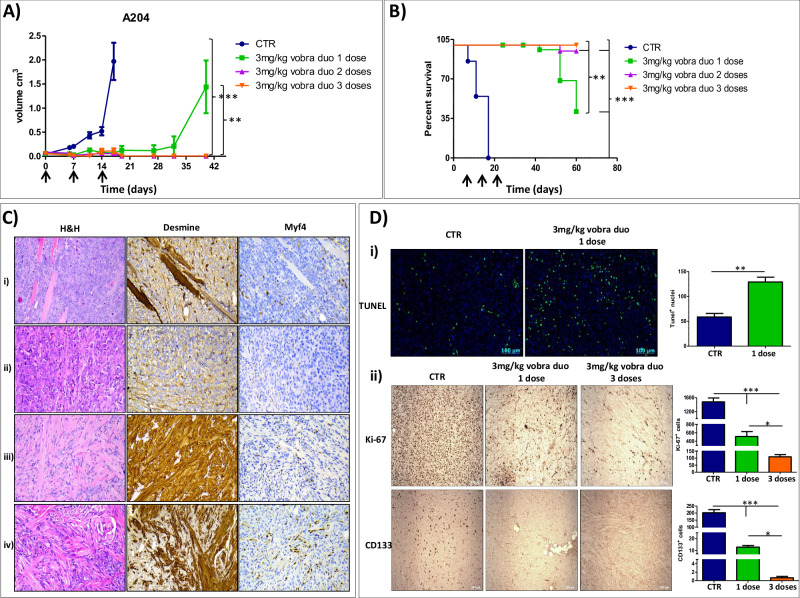
Vobra duo exerted anti-tumor effects towards a subcutaneous rhabdomyosarcoma mouse model. Mice, 7 days after being inoculated with A204 rhabdomyosarcoma cells, when tumor masses were palpable, were randomly assigned to four treatment groups and intravenously treated with PBS (CTR) or 3 mg/kg vobra duo once a week for 1, 2 or 3 weeks (vobra duo 1 dose, vobra duo 2 doses and vobra duo 3 doses, respectively). **A** Tumor growth was monitored by caliper measurement of tumor volume (*n* = 10 mice/group). Results are presented as mean ± SEM. Time was expressed in days. Day 0 represents the day of the first treatment injection, which started 7 days from tumor cell injection. **B** Survival in the subcutaneous rhabdomyosarcoma mouse model (*n* = 10 mice/group). Time was expressed in days. Day 0 represents the day of tumor cell injection. Statistical comparison of vobra duo treatments vs CTR and vs each other is reported. Black arrow: treatments. PBS, phosphate-buffered saline; vobra duo, vobramitamab duocarmazine. **C** Representative histological images showing a progressive architectural remodeling of the tumor with increasing doses of vobra duo, and the expression of differentiation markers (Desmine and Myf4). **D** Immunofluorescence TUNEL analysis (Panel **i**) and immunohystochemical Ki-67 and CD133 analysis (Panel **ii**) of subcutaneous tumors removed from vobra duo-treated and CTR mice. Original magnification 20× of a representative field for each condition was reported. Graphical quantification of the number of TUNEL, Ki-67 and CD133 positive cells was reported. Results are expressed as mean ± SD of at least five independent fields. Asterisks indicate statistical significance compared to control *p < 0.05, **p < 0.01, ***p < 0.001.

The anti-tumor efficacy of vobra duo was evaluated in a subcutaneous mouse model of rhabdomyosarcoma disease. As shown in Fig. 5A–B, vobra duo showed strong anti-tumor activity in terms of both tumor growth delay and overall survival increase, in comparison to vehicle-treated mice. Intriguingly, both tumor suppressive effects were a function of the number of administrations (A. tumor volume at day 32, CTR *vs* vobra duo 1, 2, and 3 doses, *p* < 0.001; vobra duo 1 dose *vs* vobra duo 2 and 3 doses, not statistically significant; day 40, vobra duo 1 dose *vs* vobra duo 2 and 3 doses, *p* < 0.01. B. Overall survival: CTR *vs* vobra duo 1, 2, and 3 doses, *p* < 0.001; vobra duo 1 dose *vs* vobra duo 2 and 3 doses, *p* < 0.01; vobra duo 2 doses *vs* vobra duo 3 doses not statistically significant). Importantly, no weight loss was evidenced in any of the treatment groups, including in mice treated with multiple doses of vobra duo (data not shown).

In order to highlight the impact of in vivo vobra duo administration on rhabdomyosarcoma neoplastic arrangement and the molecular mechanism implicated in the decrease of tumor volume, we performed histological analyses of A204 tumor samples. Histological evaluation of xenograft tumors revealed that repeated administration of vobra duo not only reduced tumor burden but also induced marked morphological changes in tumor architecture (Fig. 5C). Notably, after three weekly doses of vobra duo, tumor sections displayed histologic features consistent with a benign rhabdomyoma phenotype, including reduced cellular atypia, abundant eosinophilic cytoplasm, lower mitotic index, and an increased proportion of elongated, strap-like cells resembling differentiating myocytes. These features were absent or minimally represented in untreated controls or after a single dose of vobra duo (Fig. 5C). The observed shift suggests that, in addition to its cytotoxic effects, vobra duo may promote differentiation of malignant rhabdomyosarcoma cells toward a more benign muscular lineage. Immunohistochemical analyses performed on xenograft tumors after the second and the third vobra duo administration revealed a strong and diffuse expression of Desmin and Myogenin/Myf-4 (Fig. 5C, iii–iv), indicating activation of myogenic differentiation programs. These findings further support the histologic observation of a rhabdomyoma-like phenotype emerging in treated tumors. Moreover, a single dose of vobra duo, after 24 h from the injection, was able to significantly induce an increase in the number of apoptotic cells in A204 xenografts (Fig. 5D, i). The above described effects were accompanied by a pronounced reduction in the expression of proliferation (Ki-67) and stemness (CD133) markers, in a number of administration-dependent manner, further supporting the induction of a less aggressive, more differentiated tumor phenotype (Fig. 5D, ii). The enhanced expression of late myogenic markers, together with the reduction in Ki-67 and CD133, suggests that vobra duo not only exerts cytotoxic and anti-proliferative effects but may also promote lineage-specific differentiation of rhabdomyosarcoma cells toward a more mature muscular phenotype.

## Discussion

Pediatric sarcomas (pSC) are a vast group of diseases of mesenchymal origin, which continues to represent a formidable challenge for pediatric oncology, accounting for approximately 13% of cancer-related death of pediatric and young adult patients [1, 4, 32]. pSC often present as metastatic disease already at onset (about one-third of diagnosed), and localized disease frequently recurs, even after an initial response to therapy [5]. The available treatment options, consisting of a multimodal approach based on poly-chemotherapy, surgery, and/or radiotherapy, have improved the outcome of localized disease. In contrast, the prognosis for patients affected by relapsed/refractory diseases is still very poor.

The search for satisfactory treatment approaches has been pursued, and several immunotherapy-based strategies have been investigated, such as using immune-checkpoint inhibitors, adoptive CAR-T cell therapy, and cancer vaccines [33]. As of today, phase I/II clinical trials investigating the activity of PD-1 and CTLA-4 in pediatric osteosarcoma have produced disappointing results [32].

Antibody-drug conjugates, harmful moieties endowed with cytotoxic potential associated with targeting specificity, have emerged as promising therapeutic tools for treating hematological and solid malignancies, especially in adults [34]. Their hopeful application in pediatric oncology has been a matter of investigation. A mounting number of preclinical studies have focused on that [35, 36], but only a few clinical trials have aimed at evaluating the safety and efficacy of ADCs in the pediatric disease setting (clinicaltrial.gov).

It was recently reported that the investigational anti-B7-H3 antibody-drug conjugate vobramitamab duocarmazine (vobra duo) has a potent anti-tumor activity toward in vitro and in vivo preclinical neuroblastoma models [19]. Such a great result obtained in the pediatric setting encouraged us to extend the investigation to other B7-H3-expressing pediatric cancers. In this study, the anti-tumor effectiveness of vobra duo was investigated in pSC tumors, which, as already reported, share with neuroblastoma the surface expression of B7-H3 [37]. Previously reported experimental studies by the Pediatric Preclinical Testing Consortium in preclinical solid tumor models, including also sarcomas, investigated the anti-tumor activity of vobra duo and another B7-H3 targeted ADC (*ie* m276-SL-PBD), confirming the great relevance of this antigen in targeted approaches [38, 39]. Furthermore, at the beginning of 2025, the FDA has granted breakthrough therapy designation for GSK’227, also known as HS-20093 (a B7-H3 targeted ADC whose payload is a topoisomerase inhibitor), for relapsed/refractory adult osteosarcoma patients who experienced disease progression after at least two prior lines of therapy.

The results collected by the brilliant work carried out by the Pediatric Preclinical Testing Program (PPTP) before, and by its successor the Pediatric Preclinical Testing Consortium (PPTC) successively, in almost two decades of activity (2004-2021), highlighted an overestimation of efficacy for agents used in preclinical models, including ADCs, compared to the results obtained when moving to clinical trials [40]. Taking into account this observation, it sounds reasonable that the deep understanding of the underlying mechanism of action of a given ADC in the context of a specific tumor or a group of tumors can be helpful in view of the application of combination strategies. It is well documented and accepted that most of the advanced, relapsed/refractory tumors, also in the pediatric context, are subjected to treatments combining different drugs. To rationally justify drug combinations in clinical trials, solid preclinical data necessarily have to be accompanied by the knowledge of their mechanisms of action [41].

Moreover, notwithstanding the highly competitive landscape of B7-H3-targeted interventions, pediatric sarcoma remains an uncovered category worth investigating in models that have the potential to provide a rationale for clinical intervention. Further, the vast majority of B7-H3 ADCs carry topoisomerase 1 inhibitors (*eg* deruxtecan) as a payload like for example, DS-7300a and HS-20093 [42, 43]. Vobra duo payload (*ie* duocarmycin) is distinct from that of Topo inhibitors, exerting antitumor activity by suppressing proliferation as well as inhibiting translation and protein synthesis, thus addressing both proliferating cells, the primary target of Topo inhibitors, and non-proliferating cells, where Topo inhibitors are less effective.

Here, experiments performed in vitro revealed that vobra duo, like previously observed in neuroblastoma [19], exerted its anti-tumor effectiveness toward pSC, working by inhibition of cell proliferation, apoptosis induction, and cell viability reduction. Indeed, treatment with vobra duo reduced the cell viability of all the pSC cell lines analyzed when cultured in monolayer and, most importantly, as multi-cellular tumor spheroids, a model system particularly useful to closely mimic the complexity of a tumor mass and to better predict response to therapy [44, 45].

One of the most critical regulators of cellular proliferation, survival, protein synthesis, and differentiation, is the AKT/mTOR cell signaling. This pathway is commonly deregulated in cancer [46, 47], including rhabdomyosarcoma [48] and osteosarcoma [49], and it is implicated in uncontrolled tumor growth, resistance to therapies, and unfavorable prognosis [50]. Inhibition of the mTOR axis has shown promising results for the treatment of several soft tissue and bone sarcoma [51]. Our data reported that vobra duo treatment down-modulated the expression of the AKT/mTOR signaling key players, *i.e*. the phosphorylated forms of AKT, P70 S6K 4E-BP1 proteins. Interestingly, this effect was obtained, both in the more sensitive rhabdomyosarcoma A204 cells and in the more resistant osteosarcoma U-2-OS cells, by using sub-lethal doses of vobra duo, in order to avoid alteration of cellular molecular mechanisms due to the activation of necrotic cell death, which was particularly evident in the U-2-OS cell line. Interestingly, at basal conditions, the expression of the aforementioned proteins was higher, and their down-modulation by vobra duo was less significant in U-2-OS than in A204 cell line. This could in part explain why vobra duo effectiveness is more pronounced in the rhabdomyosarcoma cell line compared to the osteosarcoma one. Rescue experiments carried out by the use of IGF-1, known to be able to revert the down-modulation of the PI3K/AKT pathway [25], suggest that vobra duo led to inhibition of cell proliferation at least in part by affecting the AKT/mTOR pathway, which, as expected, isn’t the only responsible for this effect.

Noteworthy, a slight down-modulation of the total protein levels of AKT and P70 S6K was observed, likely as a compensatory mechanism triggered by prolonged and pronounced cellular stress, as a pharmacological treatment can be [52]. The sustained inhibition of the AKT-mTOR pathway may lead to altered expression or reduced stability of key pathway components, such as AKT and P70S6K. Under stress conditions, cells attempt to survive by minimizing energy consumption, which includes down-regulating energy-intensive processes like protein synthesis. By contrast, the increase in the total level of 4E-BP1 may reflect a compensatory response. Since the phosphorylated (inactive) form of 4E-BP1 is markedly reduced, cells might upregulate the total protein levels to re-establish control over cap-dependent translation and maintain protein synthesis under stress [53]. These responses are consistent with tumor cell behavior, where genetic alterations drive continuous proliferation and survival, even under drug-induced stress [54]. Importantly, the ratio between phosphorylated and total forms of each protein clearly and significantly demonstrates the inhibition (“switching off”) of the AKT/mTOR pathway.

Mechanistic investigations highlighted that in the pSC models, vobra duo induced apoptotic cell death not only in a caspase-dependent manner but also through a caspase-independent pathway. Different from what was reported for the neuroblastoma model [19], the abrogation of caspase activity, by using a pan-caspase inhibitor, only partially reverted vobra duo-induced apoptosis in pSC cells. This suggests that caspase-independent cell death could be induced by altered biological features such as mitochondrial dysfunction [55]. Mitochondria deregulation represents a double-edged sword in cancer, including sarcoma malignances [56]. Mitochondrial stress could explicate a pro-tumorigenic effect, by altering tumor metabolism and increasing ROS production. ROS activates pro-survival pathways such as AKT/mTOR signaling leading to uncontrolled tumor cell proliferation, metastatization, and drug-resistance [57]. By the way, anti-cancer therapies can damage DNA and exacerbate ROS production beyond the oxidative stress threshold, leading to cell death [55]. Vobra duo treatment caused a strong mitochondrial fitness disruption in all pSC cell lines, by depolarizing mitochondrial membrane potential, increasing mitochondrial superoxide production, and reducing ATP synthesis trough FoF1 ATP synthase. These metabolic alterations lead to the cellular energy status impairment and increase in the anaerobic glycolysis, probably as an attempt to compensate the altered mitochondrial function. All these events finally culminate in the activation of Apoptosis Inducible Factor (AIF) protein, which triggers caspase-independent cell death through chromatin condensation and DNA fragmentation [31]. AIF is a flavoprotein physiologically expressed and localized mainly within mitochondria, where it supports mitochondrial functions, by maintaining electron transport chain activity, reducing oxidative stress, and preventing excessive ROS production. In cellular stress conditions, AIF can translocate to the nucleus, leading to cell death. Our results showed that vobra duo treatment determined the translocation of AIF, already expressed in the cytosol at the basal level in all pSC cell lines, to the nuclei, confirming implication in caspase-independent cell death [31, 58, 59]. Noteworthy, in the U-2-OS cell line, which is the most treatment-resistant, AIF seems not to be implicated in apoptotic cell death, and this behavior seems to be in line with its more pronounced tendency to undergo necrosis than apoptotic cell death.

Our findings agree with previously published papers reporting that ADCs can induce mitochondria-dependent apoptosis [60, 61]. In particular, a TROP2-targeted ADC, used in a pancreatic cancer model, led to mitochondria-related apoptosis [60]. Recently, it was also elucidated the mechanism responsible for hepatotoxicity of Ado-Trastuzumab Emtansine (TDM-1), which is an HER-2 targeted ADC, approved by the FDA to treat metastatic breast cancer. Mechanistic investigations revealed that the observed hepatotoxicity was related to TDM-1-mediated mitochondria-dependent apoptosis induction in hepatocytes [61].

Finally, a xenograft rhabdomyosarcoma mouse model, developed via subcutaneous inoculum of A204 cells, contributed to confirming and widening the anti-tumor activity of vobra duo in the pediatric cancer *scenario*. A treatment schedule consisting of a weekly intravenous administration of vobra duo was effective in the A204 mouse model, in a dose-dependent fashion, in terms of both tumor growth delay and increased survival.

Great interest exists in evolving new therapeutic strategies that facilitate rhabdomyosarcoma tumors to terminally differentiate into non-proliferating muscle cells [62]. Of particular relevance, the observed histological transition from rhabdomyosarcoma to rhabdomyoma following repeated vobra duo administration suggests a differentiation-inducing effect, which may represent a novel therapeutic avenue aimed at reprogramming tumor aggressiveness rather than solely eliminating tumor cells. Our findings demonstrate that treatment with vobramitamab duocarmazine induces a morphologic and immunophenotypic shift of rhabdomyosarcoma cells toward a rhabdomyoma-like phenotype, as evidenced by the strong expression of Desmin and Myogenin/Myf-4 after the second dose. This differentiation-associated response supports the hypothesis that, beyond cytotoxicity, vobra duo may promote a partial restoration of myogenic lineage commitment. Such an effect is consistent with prior observations of post-therapy rhabdomyoblast differentiation in embryonal rhabdomyosarcoma, where Desmin and Myogenin expression increase following cytotoxic treatment [63]. Mechanistically, several studies have demonstrated that rhabdomyosarcoma cells exhibit a block in terminal myogenic differentiation due to deregulation of myogenic regulatory factors (MRFs) and altered epigenetic control [64, 65]. Restoration of MyoD–E-protein heterodimer function can overcome this block and induce differentiation, suggesting that pharmacologic agents capable of reactivating these transcriptional networks may promote tumor maturation [66]. Moreover, recent data indicate that sustained TRPS1 expression represses MYOG transcription and that TRPS1 downregulation restores terminal differentiation in embryonal RMS [67]. Collectively, these findings align with our observation of enhanced Desmin and Myf-4 expression following vobra duo exposure, implying that the drug may alleviate differentiation blockade through modulation of transcriptional or epigenetic regulators. Similar to the differentiation observed upon MET receptor inhibition, which reduces RMS aggressiveness and metastatic potential [68], vobra duo may exert part of its antitumor efficacy by promoting a more mature, less proliferative myogenic phenotype.

Moreover, in A204 xenografts, vobra duo treatment caused a severe down-modulation of the expression of the cancer stem cell marker CD133. CD133, also named prominin-1, is a membrane glycoprotein commonly associated with a more aggressive and chemotherapy-resistant tumor phenotype. Its expression is found in various types of cancer, including rhabdomyosarcoma [69, 70]. Since CD133 expression prevented tumor cells differentiation, for instance, in neuroblastoma [71], the observed down-modulation of CD133 in A204 xenografts, after vobra duo treatment, could be associated with the aforementioned capability of vobra duo to induce rhabdomyosarcoma differentiation toward rhabdomyoma. This observed effect could open the possibility of establishing new therapeutic combinations to increase sarcoma responsiveness to treatments, minimizing the doses of the single agents and maximizing their anti-cancer effect. Given a hopeful clinical translation, it is to be underlined that two doses of vobra duo proved as effective as three doses, in the treatment of the rhabdomyosarcoma mouse model. This result is of particular interest and suggests the possibility of using a lower total dose treatment schedule to prevent potential side effects without affecting the anti-cancer efficacy in future and desirable clinical settings.

## Conclusion

Overall, this study demonstrated that vobra duo exerts an anti-tumor activity towards pSC preclinical models in vitro and in vivo. In vitro, from a mechanistic point of view, vobra duo acts by reducing cell viability, inhibiting cell proliferation, and inducing caspase-dependent and caspase-independent apoptosis. In the xenograft rhabdomyosarcoma mouse model, vobra duo led to tumor growth reduction and increased life span, by determining, besides the reduction of tumor cell proliferation index, the down-modulation of tumor cells staminality phenotype, as well as by differentiating rhabdomyosarcoma tumor cells from their malignant phenotype to the benign phenotype of the soft tissue tumor rhabdomyoma. These encouraging results highlight the need of extending the anti-tumor efficacy of vobra duo toward a panel of patient-derived preclinical models to pave the way for clinical translation.

## Supplementary information

Supplementary Figure 1

Supplementary Figure 2

Supplementary Figure 3

Supplementary Figure 4

Legends to Supplementary Figures

## Data Availability

Data are available upon kind request.
