# Supplementary figures and images for: The investigational anti-B7-H3 antibody-drug conjugate vobramitamab duocarmazine exerts anti-tumor activity in vitro and in vivo in pediatric sarcoma preclinical models
Source: Cell Death Dis. 2026 Jan 8;17(1):173. doi: 10.1038/s41419-025-08397-z (PMC12877178; doi:10.1038/s41419-025-08397-z)

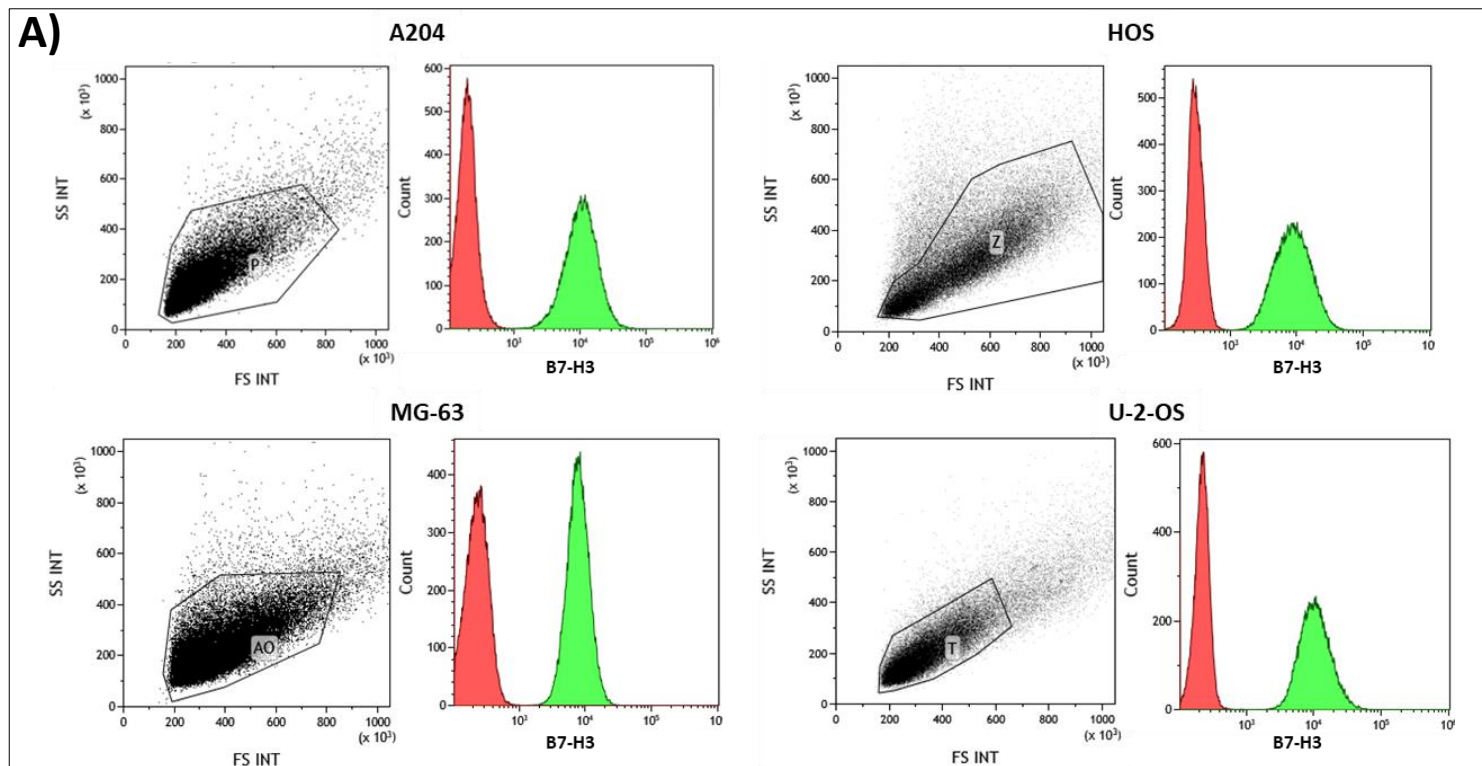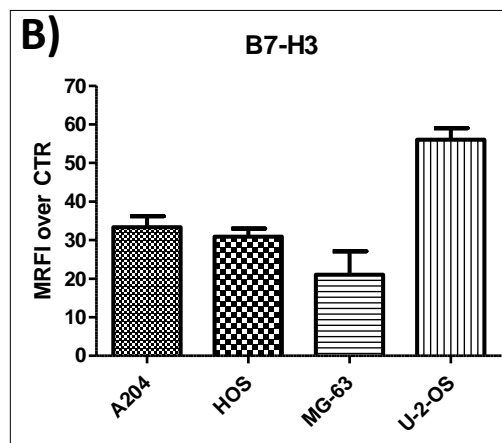

Supplementary Figure 1

Supplement: Supplementary file 1 — Supplementary Figure 1 [file 41419_2025_8397_MOESM1_ESM.pdf]

A)

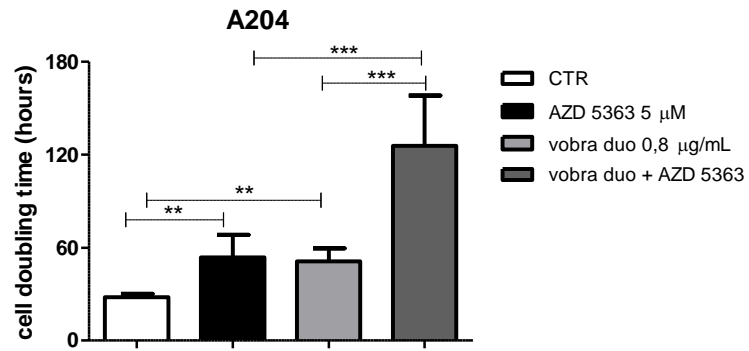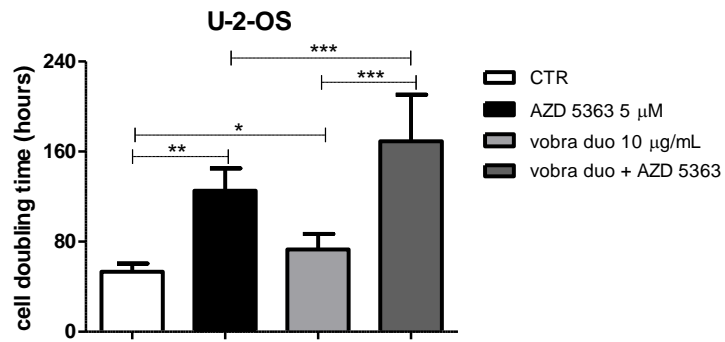

B)

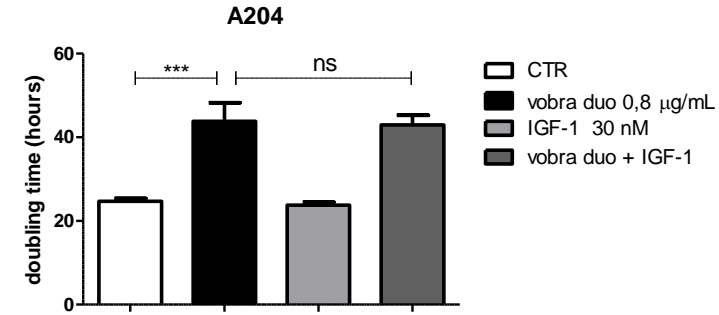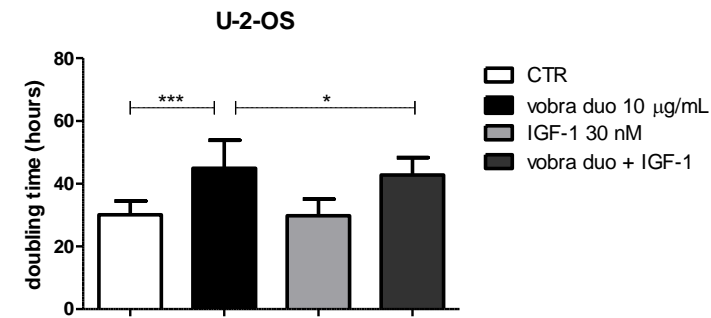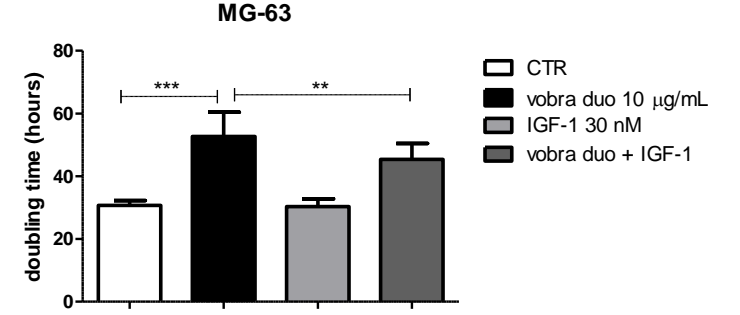

Supplement: Supplementary file 2 — Supplementary Figure 2 [file 41419_2025_8397_MOESM2_ESM.pdf]

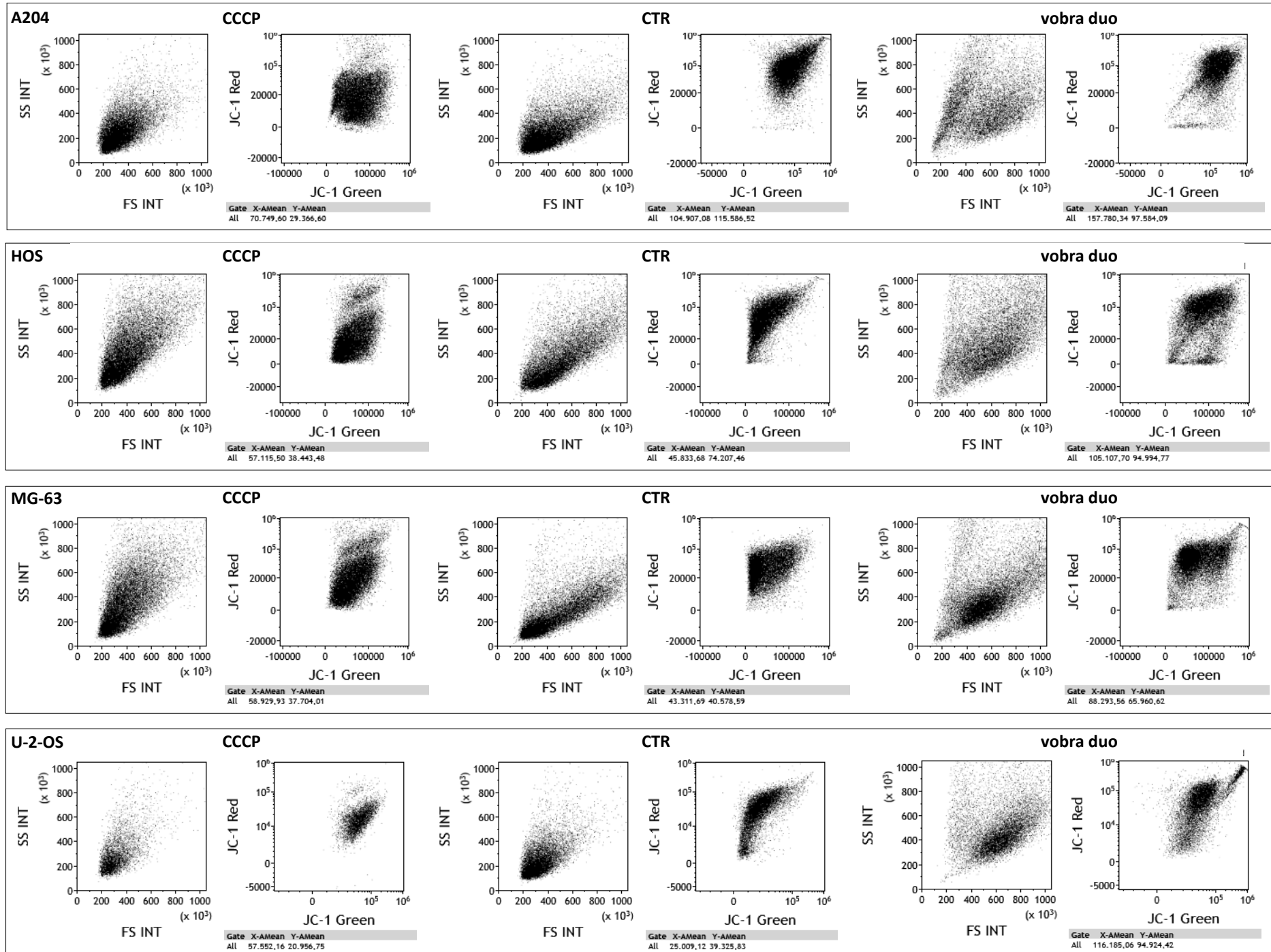

**Supplementary Figure 3**

Supplement: Supplementary file 3 — Supplementary Figure 3 [file 41419_2025_8397_MOESM3_ESM.pdf]

A)

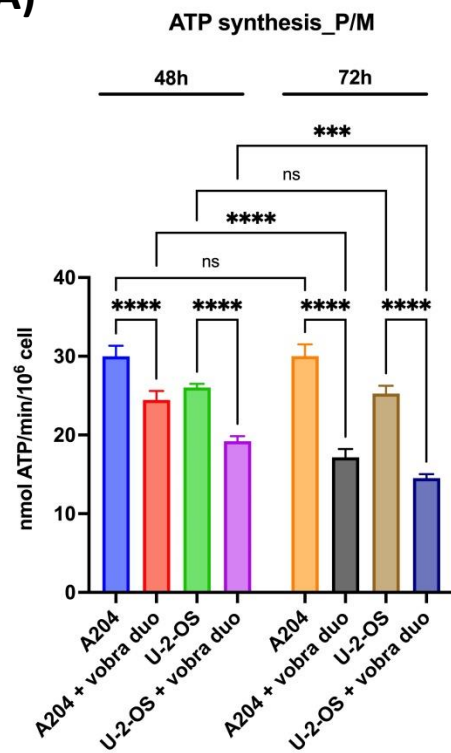

B)

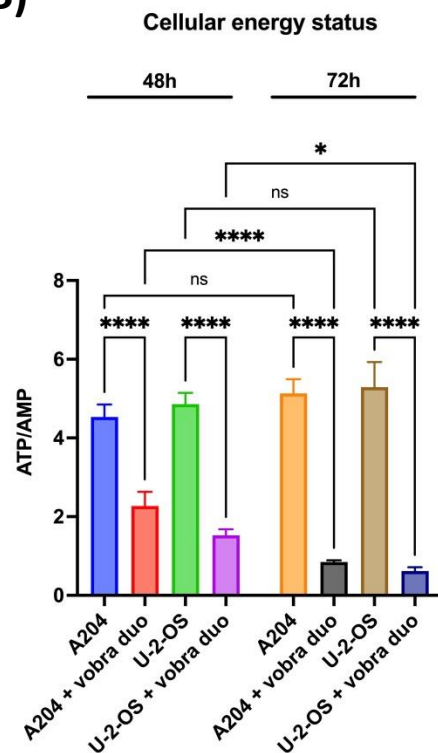

C)

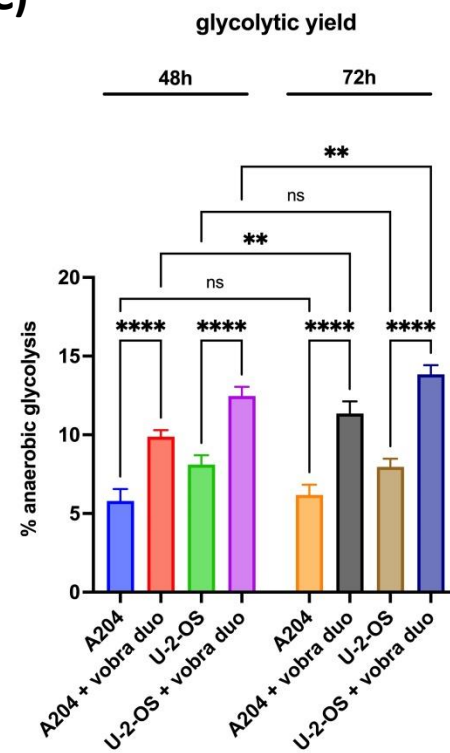

Supplement: Supplementary file 4 — Supplementary Figure 4 [file 41419_2025_8397_MOESM4_ESM.pdf]
