# Supplementary material for: The investigational anti-B7-H3 antibody-drug conjugate vobramitamab duocarmazine exerts anti-tumor activity in vitro and in vivo in pediatric sarcoma preclinical models
Source: Cell Death Dis. 2026 Jan 8;17(1):173. doi: 10.1038/s41419-025-08397-z (PMC12877178; doi:10.1038/s41419-025-08397-z)
Supplement: Supplementary file 5 — Legends to Supplementary Figures [file 41419_2025_8397_MOESM5_ESM.pdf]

Legends to Supplementary Figures

Supplementary Figure 1: B7-H3 expression by human pSC cell lines. A) Representative flow cytometry histograms of B7-H3 expression in human rhabdomyosarcoma (A204) and osteosarcoma (HOS, MG-63 and U-2-OS) cell lines. B7-H3 PE: PE- conjugated anti-human B7-H3 mAb. Green: cells labeled with PE-conjugated mouse IgG1 anti-B7-H3 mAb. Red: cells labeled with isotype-matched mouse IgG1 control mAb. **B)** The histograms represent the expression of human B7-H3 by human pSC cell lines. Protein expression was evaluated by flow cytometry and is expressed as mean ratio fluorescence intensity (MRFI) of positive labeled cells over cells stained with an isotype matched control Ab.

Supplementary Figure 2: Involvement of the AKT/mTOR pathway in the anti-proliferative effects of vobra duo. A204 and U-2-OS cell lines were exposed to treatment with AZD 5363 (5µM), and IGF-1 (30 nM), alone and in combination with vobra duo (at the concentration indicated in the Figure). For IGF-1, a pre-treatment of 30 minutes at 37 °C was carried out. Cell doubling time was measured thank to a ptychography-based single cell segmentation analysis, performed in time-lapse for 72 hours. Every experimental condition was carried out in sextuplicate, and experiments were repeated twice. * $p<0.05$, ** $p<0.01$, *** $p<0.001$.

Supplementary Figure 3: Mitochondrial membrane depolarization observed in pSC cells treated with or without vobra duo

Change in JC-1 green and red fluorescence in pSC cells treated *in vitro* with vobra duo (0,8 µg/mL for A204, 8 µg/mL for HOS and MG-63 and 16 µg/mL U-2-OS) continuously administered for 48h hours, was detected by flow cytometer. JC-1 is a probe, which accumulates within the cell in a mitochondria membrane potential-dependent fashion. It assembles red fluorescent aggregates (590 nm; red) in polarized mitochondria, while it monomerizes in depolarized mitochondria, emitting a green fluorescence (527 nm; green). Compensation was done using Kaluza software (Beckman Coulter Inc.) and logarithmic scales were used to display better both parameters. CCCP: internal positive control; CTR: untreated cells; vobra duo: vobra duo-treated cells.

Supplementary Figure 4: ATP synthesis, cellular energy status, and glycolytic yield in A204 and U-2-OS cell lines treated with vobra duo.

All data were obtained from A204 and U-2-OS cell lines untreated or treated with vobra duo for 48 and 72 h. (**A**) Aerobic ATP synthesis stimulated by pyruvate plus malate (P/M). (**B**) Cellular energy status calculated as the ratio between the intracellular contents of ATP and AMP. (**C**) Glycolytic yield, calculated as the percentage of the actual lactate release (data from panel B) relative to the theoretical lactate production obtained as the double of consumed glucose (data from panel **A**), assuming that two lactate molecules are generated from one molecule of glucose during anaerobic glycolysis. Data are representative of three independent experiments (n= 3) and are expressed as mean ± SD. Statistical analysis was performed by one-way ANOVA followed by Dunnett's multiple comparisons test. * $p < 0.05$, ** $p < 0.01$, ***$p < 0.001$, and ****$p < 0.0001$. ns indicates a no significant difference.
